# The SMARCD Family of SWI/SNF Accessory Proteins Is Involved in the Transcriptional Regulation of Androgen Receptor-Driven Genes and Plays a Role in Various Essential Processes of Prostate Cancer

**DOI:** 10.3390/cells12010124

**Published:** 2022-12-28

**Authors:** Iris E. Ertl, Robert Brettner, Hannah Kronabitter, Thomas Mohr, Sophia Derdak, Markus Jeitler, Martin Bilban, Nathalie Garstka, Shahrokh F. Shariat

**Affiliations:** 1 Department of Urology, Comprehensive Cancer Center, Medical University of Vienna, 1090 Vienna, Austria; 2 Center for Cancer Research, Comprehensive Cancer Center, Medical University of Vienna, 1090 Vienna, Austria; 3 Department of Analytical Chemistry, Faculty of Chemistry, University of Vienna, 1010 Vienna, Austria; 4 Joint Metabolome Facility, University of Vienna and Medical University Vienna, 1090 Vienna, Austria; 5 Core Facilities, Medical University of Vienna, 1090 Vienna, Austria; 6 Department of Laboratory Medicine, Medical University of Vienna, 1090 Vienna, Austria; 7 Department of Urology, Weill Cornell Medical College, New York, NY 10065, USA; 8 Department of Urology, University of Texas Southwestern, Dallas, TX 75390, USA; 9 Department of Urology, Second Faculty of Medicine, Charles University, 150 06 Prag, Czech Republic; 10 Hourani Center for Applied Scientific Research, Al-Ahliyya Amman University, Amman 19328, Jordan

**Keywords:** prostate cancer, chromatin-remodeling, SWI/SNF complex, SMARCD1/BAF60A, SMARCD2/BAF60B, SMARCD3/BAF60C

## Abstract

Previous studies have demonstrated an involvement of chromatin-remodelling SWI/SNF complexes in the development of prostate cancer, suggesting both tumor suppressor and oncogenic activities. SMARCD1/BAF60A, SMARCD2/BAF60B, and SMARCD3/BAF60C are mutually exclusive accessory subunits that confer functional specificity and are components of all known SWI/SNF subtypes. To assess the role of SWI/SNF in prostate tumorigenesis, we studied the functions and functional relations of the SMARCD family members. Performing RNA-seq in LnCAP cells grown in the presence or absence of dihydrotestosterone, we found that the SMARCD proteins are involved in the regulation of numerous hormone-dependent AR-driven genes. Moreover, we demonstrated that all SMARCD proteins can regulate AR-downstream targets in androgen-depleted cells, suggesting an involvement in the progression to castration-resistance. However, our approach also revealed a regulatory role for SMARCD proteins through antagonization of AR-signalling. We further demonstrated that the SMARCD proteins are involved in several important cellular processes such as the maintenance of cellular morphology and cytokinesis. Taken together, our findings suggest that the SMARCD proteins play an important, yet paradoxical, role in prostate carcinogenesis. Our approach also unmasked the complex interplay of paralogue SWI/SNF proteins that must be considered for the development of safe and efficient therapies targeting SWI/SNF.

## 1. Introduction

Human SWI/SNF complexes represent a heterogeneous family of ATP-dependent chromatin remodelers consisting of 12–15 subunits [1]. These complexes are able to modulate the accessibility of a given genomic region, thereby acting as transcriptional regulators [2]. SWI/SNF complexes are involved in different cellular processes including differentiation, chromosomal stability and DNA repair [3,4,5,6]. Mutations in SWI/SNF genes are present in ~20% of human cancers [7,8]. Although mutations of SWI/SNF subunits are relatively infrequent in prostate cancer (PCa), previous studies have suggested that SWI/SNF complexes can promote prostate tumourigenesis [9]. Increased SMARCA4/BRG1 expression, for instance, has been shown to be associated with tumour development and invasiveness, while PBMR1/BAF180 was identified as a driver of progression to the castration-resistant state [10,11]. SMARCA4/BRG1 has also been shown to play a role in the biologic aggressiveness of neuroendocrine prostate cancer (NEPC) [12]. Moreover, a recent study reported that the novel drug AU-15330, which specifically inhibits SMARCA4/BRG1, SMARCA2/hBRM and PBRM1/BAF180, has potent inhibitory functions on enhancer-addicted PCa [13]. On the other hand, the literature suggests tumour suppressor functions to SWI/SNF complexes in prostate tissue. Amongst others, it was reported that the long noncoding RNA (lncRNA) SChLAP1 promotes aggressive PCa through antagonizing the tumour suppressor activity of SMARCB1/BAF47 [14]. Moreover, SMARCA2/hBRM has been demonstrated to have anti-proliferative functions and to be protective against the progression to castration-resistance, while BRG1/SMARCA4 is required for the repressive action of the tumour suppressor prohibitin [15,16]. 

The accessory SWI/SNF subunit SMARCD1/BAF60A represents a cofactor of the androgen receptor (AR) that selectively activates AR-driven genes [17]. SMARCD1 was identified as a target of the miR-99 family members of microRNAs that have been shown to suppress the proliferation of PCa cells, suggesting oncogenic functions [18]. Like other accessory subunits, SMARCD1 and its highly similar and mutually exclusive paralogues, SMARCD2/BAF60B and SMARCD3/BAF60C, are thought to confer specificity to a given SWI/SNF complex [19]. SMARCD proteins are incorporated in all known SWI/SNF subtypes, i.e., canonical BRG1/BRM-associated factor (cBAF), polybromo-associated BAF (PBAF), and recently described non-canonical BAF (ncBAF) complexes [20]. Thus, to obtain a comprehensive overlook of SWI/SNF in prostate tumorigenesis, we decided to study the SMARCD proteins in cellular models of this highly prevalent malignancy.

Here, we demonstrate that SMARCD1, SMARCD2 and SMARCD3 exhibit independent, shared and redundant functions in prostate cells, and are crucial factors for cellular processes such as the maintenance of cellular morphology and cytokinesis. We further show that all SMARCD proteins are involved in hormone-dependent AR-driven signaling pathways, but also exhibit regulatory effects antagonizing AR-signaling. Moreover, we demonstrate that the SMARCD proteins can act in a hormone-independent manner and are able to regulate AR-driven target genes in androgen-depleted cells. 

## 2. Materials and Methods

### 2.1. Cell Lines 

The cell lines RWPE-1, LnCAP, C4-2, PC3, and DU145 were purchased from ATCC (Manassas, VA, USA). Except of RWPE-1, all cells were cultivated in RPMI 1640 medium (Gibco™ RPMI 1640 Medium, Thermo Fisher Scientific, Waltham, MA, USA) supplemented with 10% fetal bovine serum (FBS) (Gibco™ Fetal Bovine Serum, Thermo Fisher Scientific) or charcoal stripped FBS (Gibco™ One Shot™ FBS, Thermo Fisher Scientific) supplemented with defined concentrations of DHT (0.1 nM–100 nM) (Sigma-Aldrich, St. Louis, MO, USA). RWPE-1 cells were cultured in keratinocyte serum-free medium containing 5 ng/mL epidermal growth factor (EGF) and 50 μg/mL bovine pituitary extract (BPE) (Gibco™ Keratinocyte SFM, Thermo Fisher Scientific), that was supplemented with 1 nM DHT (Sigma-Aldrich) if required. All cell lines were cultivated under standard conditions (37 °C; 5% CO_2_). The *mycoplasma* contamination status was monitored using the MycoAlert™ Mycoplasm Detection Kit (Lonza, Basel, Switzerland). 

### 2.2. Quantitative Polymerase Chain Reaction (qPCR) 

RNA was isolated using TRIzol™ Reagent (Thermo Fisher Scientific, Waltham, MA, USA) and cDNA was generated with TaqMan™ Reverse Transcription Reagents (Thermo Fisher Scientific). Primers were designed to span one exon-exon junction and were purchased from Eurofins Austria (Wiener Neudorf, Austria). qPCR was performed in experimental triplicates using SYBR™ Green PCR Master Mix (Thermo Fisher Scientific) and the QuantStudio™ 7 Flex Real-Time PCR System (Thermo Fisher Scientific). GAPDH and 18sRNA served as housekeeping genes.

### 2.3. Western Blotting 

Protein extracts were generated with RIPA lysis buffer (ab156034, Abcam, Cambridge, UK) supplied with protease inhibitors (cOmplete^™^ Mini Protease Inhibitor Cocktail Tablets, Roche, Basel, CH). Protein concentrations were determined using the Pierce™ BCA Protein Assay Kit (Thermo Fisher Scientific, Waltham, MA, USA). Western blotting was performed using 10% Mini-PROTEAN^®^ TGX Stain-Free™ Protein Gels and the TransBlot^®^ Turbo^TM^ Transfer System (Bio-Rad Laboratories, Hercules, CA, USA). Membranes were blocked in 5% milk or BSA solution for 1 h at RT and incubated with the respective antibodies (α-SMARCD1: sc-135843, Santa Cruz Biotechnologies, Dallas, TX, USA; α-SMARCD2: 2F7, Novus Biologicals, Littleton, CO, USA; α-SMARCD3: 12838-1-AP, Proteintech Group, Rosemont, IL, USA) overnight at 4 °C. Incubation with secondary antibodies (goat anti-rabbit IgG H&L (HRP), ab6721, Abcam; goat anti-mouse IgG H&L (HRP), ab6789, Abcam) was performed for 1 h at RT. Membranes were developed using Pierce™ ECL Western Blotting Substrate (Thermo Fisher Scientific).

### 2.4. siRNA-Mediated Knock-Down

Using RNAiMAX Transfection Reagent (Thermo Fisher Scientific, Waltham, MA, USA), LnCAP and RWPE-1 cells were transfected with 15 nM siRNAs targeting SMARCD1 (sc-72598, Santa Cruz Biotechnologies; s13152, Thermo Fisher Scientific), SMARCD2 (sc-93762, Santa Cruz Biotechnologies; s13154, Thermo Fisher Scientific), SMARCD3 (sc-89355, Santa Cruz Biotechnologies; s13159; Thermo Fisher Scientific), AR (s1538; Thermo Fisher Scientific), or a scrambled control (Silencer^TM^ Select Negative Control No.1, Thermo Fisher Scientific). For double knockdown experiments, cells were transfected with a pool of two specific siRNAs or one specific siRNA and the scrambled control to ensure a concentration of 30 nM siRNA across all samples. 

### 2.5. Cell Viability Assays 

LnCAP cells were seeded in 96-well plates and allowed to adhere for 24 h. Single and double knockdown of SMARCD1, SMARCD2 and SMARCD3 was performed in experimental quadruplicates. After five days, cell viability was measured using the CellTiter-Glo^®^ Luminescent viability assay (Promega, Madison, WI, USA).

### 2.6. Immunofluorescence Staining 

LnCAP or RWPE-1 cells were seeded in glass chamber slides and siRNA was performed as described above. After five days, cells were fixated with 1.6% paraformaldehyde, blocking was performed using 5% goat serum (Abcam, Cambridge, UK) and 0.3% Triton X-100 (Sigma-Aldrich, St. Louis, MO, USA) in TBS. Cells were incubated with primary antibodies targeting β-tubulin (9F3; Cell Signalling Technology, Danvers, MA, USA) or nuclear pore complex proteins (Mab414, Abcam) o/n at 4 °C. Incubation with the secondary antibodies (Alexa Fluor^®^ 488 Goat anti-rabbit, Abcam; Alexa Fluor^®^ 546 Goat anti-mouse, Invitrogen) was performed for 1 h at RT. Before mounting the samples with Fluoroshield^TM^ (Sigma-Aldrich), DAPI staining was performed for 3 min at RT. Images were taken using a NIKON C2 Eclipse Ti microscope (Nikon, Tokyo, Japan).

### 2.7. RNA-Sequencing and Data Processing 

LnCAP cells were cultivated in RPMI 1640 medium (Gibco™ RPMI 1640 Medium, Thermo Fisher Scientific, Waltham, MA, USA) supplemented with 10% charcoal stripped FBS (Gibco™ One Shot™ FBS, Thermo Fisher Scientific) and siRNA was performed in triplicates as described above. After 48 h, cells were supplied with fresh androgen-depleted medium with or without 1 nM DHT (Sigma-Aldrich, St. Louis, MO, USA) and incubated for 24 h. Sequencing libraries were prepared at the Core Facility Genomics, Medical University of Vienna, using the NEBNext Poly(A) mRNA Magnetic Isolation Module and the NEBNext UltraTM II Directional RNA Library Prep Kit for Illumina (New England Biolabs, Ipswich, MA, USA). Libraries were QC-checked on a Bioanalyzer 2100 (Agilent Technologie, Santa Clara, CA, USA) using a High Sensitivity DNA Kit for correct insert size and quantified using Qubit dsDNA HS Assay (Thermo Fisher Scientific). Pooled libraries were sequenced on two flowcells of a NextSeq500 instrument (Illumina, San Diego, CA, USA) in 1x75 bp single-end sequencing mode. Per sample, on average, 25 million reads were generated. Reads in fastq format were generated using the Illumina bcl2fastq command line tool (v2.19.1.403) (llumina, San Diego, CA, USA) including trimming of the sequencing adapters. Reads in fastq format were aligned to the human reference genome version GRCh38 with Gencode 29 annotations using STAR aligner version 2.6.1a in 2-pass mode and raw reads per gene were counted by STAR [21,22,23]. Differential gene expression was calculated using DESeq2 version 1.22.2 [24]. TPM were generated by RSEM [25]. Differential gene expression was considered statistically significant for genes with log2 fold changes > ±1 and adjusted *p*-values < 0.05. Volcano Plots were created using the VolcaNoseR web app [26]. Pathway enrichment analysis was performed using the analysis platform InnateDB [27]. Gene Set Variation Analysis (GSVA) was performed using the "gsva" R-package, the classes C2- and C5- biological processes of the molecular signature database were used as input [28,29]. Differentially enriched pathways and GO terms were determined using the R-package “LIMMA” [30].

## 3. Results

### 3.1. SMARCD Genes Are Altered in Considerable Fractions of PCa Patients

**Figure 1:**
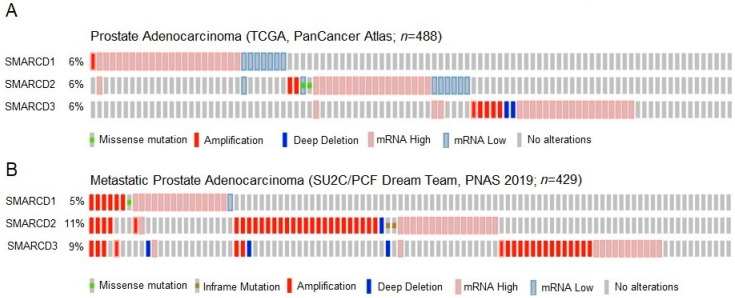
Alterations of SMARCD1, SMARCD2 and SMARCD3 in two PCa patient cohorts. (**A**) SMARCD1, SMARCD2 and SMARCD3 were each found altered in 6% of patients (*n* = 488) suffering from non-metastatic prostate adenocarcinoma of various stages. While mutations were infrequent, the majority of alterations accounted for mRNA up-regulation. (**B**) In a cohort of patients with metastatic disease (*n* = 429), alterations of SMARCD1, SMARCD2 and SMARCD3 were detected in 5%, 11% and 9% of cases, respectively. The predominant types of alterations were mRNA up-regulation and gene amplifications.

Using the cBioPortal, we assessed alterations of the SMARCD genes in two clinical data sets (Figure 1) [31,32]. Alterations of SMARCD1, SMARCD2 or SMARCD3 were each found in 6% of a patient cohort suffering from non-metastatic PCa of various clinical stages (*n* = 488; T2a-T4) (Figure 1A). In agreement with previous data, we found that mutations of the SMARCD genes were comparably infrequent in PCa (Figure S1) [9]. As in many other human malignancies, the most prevalent type of alteration was mRNA upregulation (Figure 1A and Figure S1). In a cohort of metastatic patients (*n* = 429), SMARCD1, SMARCD2 or SMARCD3 were altered in 5%, 11%, and 9% of cases, respectively (Figure 1B). The vast majority of alterations accounted for mRNA up-regulation and gene amplifications (Figure 1B).

### 3.2. SMARCD3 Expression Levels Are Increased in Malignant Prostate Cell Lines 

**Figure 2:**
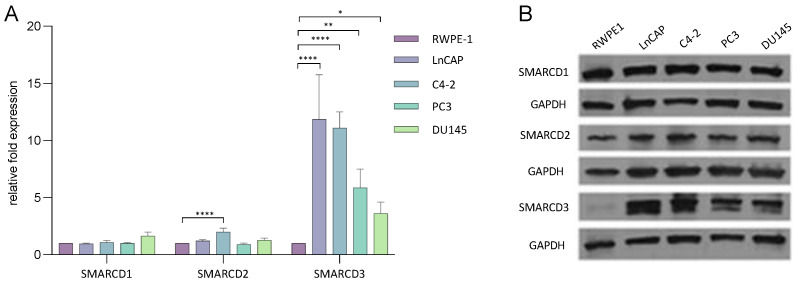
mRNA and protein levels of SMARCD1, SMARCD2 and SMARCD3 in benign and malignant prostate cell lines. (**A**) Expression levels of SMARCD1, SMARCD2 and SMARCD3 in various prostate cell lines were measured by qPCR. RNA was isolated from several biological replicates (*n* = 3). Asterisks indicate the statistical significance of differences in gene expression between the respective cell line and non-malignant RWPE-1 cells. Error bars indicate the standard error of the mean (SEM). Standard deviations were calculated using the relative-fold expression determined in various technical replicates (*n* = 6). * *p* ≤ 0.05; ** *p* ≤ 0.01; *** *p* ≤ 0.001; **** *p* ≤ 0.0001. (**B**) SMARCD1, SMARCD2 and SMARCD3 protein levels in various prostate cell lines were determined by Western blotting.

SMARCD1, SMARCD2 and SMARCD3 expression levels were assessed in the cell lines RWPE-1, LnCAP, C4-2, PC3 and DU145 cultivated with 1 nM 5α-dihydrotestosterone (DHT) (Table 1 and Figure 2A). 

Compared to non-malignant RWPE-1 cells, SMARCD1 and SMARCD2 levels were not or only slightly altered in the PCa cell lines (Figure 2A). SMARCD3 exhibited clearly higher expression levels in all PCa cell lines compared to RWPE-1 (Figure 2A). Western blotting confirmed comparable SMARCD1 and SMARCD2 protein levels across all cell lines, and clearly elevated SMARCD3 levels in malignant cells (Figure 2B). Comparing the SMARCD genes amongst each other, SMARCD1 exhibited the highest expression across all studied cell lines (Figure S2).

### 3.3. SMARCD3 Is an Androgen-Regulated Gene 

**Figure 3:**
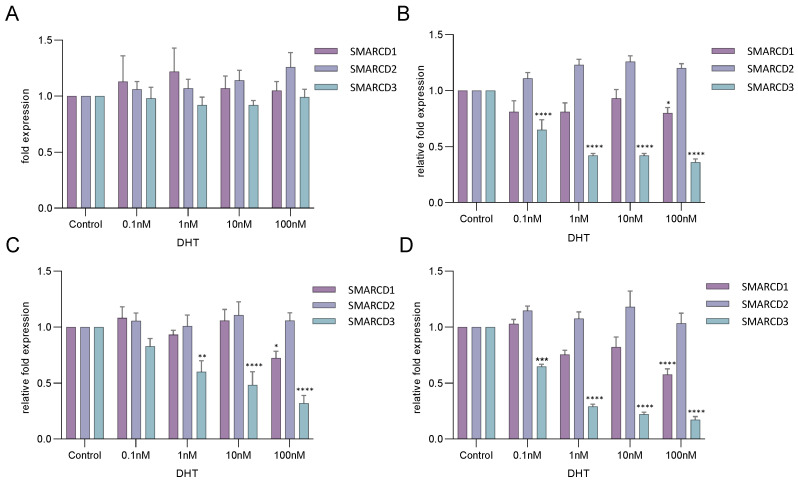
SMARCD3 is an androgen-regulated gene. LnCAP cells were induced with various concentrations of DHT for (**A**) 8 h, (**B**) 24 h, (**C**) 48 h or (**D**) 72 h and SMARCD1, SMARCD2 and SMARCD3 expression levels were measured by qPCR. RNA was isolated from several biological replicates (*n* = 3). Asterisks indicate the statistical significance of differences in gene expression compared to the androgen-deprived control. Error bars indicate the standard error of the mean (SEM). Standard deviations were calculated using the relative-fold expression determined in various technical replicates (*n* = 6). * *p* **≤** 0.05; ** *p* **≤** 0.01; *** *p* ≤ 0.001; **** *p* ≤ 0.0001.

SMARCD1, SMARCD2 and SMARCD3 expression levels were determined in LnCAP cells treated with various concentrations of DHT (Figure 3). While no expression changes were observed after 8 h (Figure 3A), SMARCD3 was downregulated in a concentration-dependent manner after prolonged incubation periods (Figure 3B–D). Incubation with 100 nM DHT for 24 h, 48 h and 72 h caused a decrease of SMARCD3 levels to 36%, 32% and 17% compared to the androgen-deprived control, respectively (Figure 3B–D). DHT also induced down-regulation of SMARCD1, however, the effect was far less pronounced (Figure 3B–D). SMARCD2 levels were not significantly altered in response to androgens (Figure 3A–D). Previous studies demonstrated that direct AR target genes exhibited differential expression already 4 h after androgen induction, while expression changes of indirectly regulated genes were observable after 16 h to 24 h [39,40]. The delayed response of SMARCD3 to DHT, thus, suggests that the gene represents an indirect target of AR. Performing siRNA-mediated knockdown of AR in LnCAP cells, we found the direct AR-targets *KLK3* and *TMPRSS2* massively down-regulated after 24 h, while SMARCD3 levels were not altered (Figure S3) [41,42]. AR/siRNA applied for 48 h and 72 h caused a significant increase of SMARCD3 expression, thus, supporting this hypothesis (Figure S3).

### 3.4. The SMARCD Proteins Are Required for Cell Viability, The Maintenance of Cellular Morphology and Correct Cell Division 

To evaluate functional relations, SMARCD1, SMARCD2 and SMARCD3 were knocked down in various combinations and cell viability was determined after five days. Considering the high similarity of the SMARCD family members (57–72% at amino acid level), the specificity and efficiency of the respective siRNAs were carefully evaluated (Figures S4 and S5). Since it was previously shown that knockdown of AR causes growth inhibition in PCa cell lines, AR/siRNA was performed as a positive control [43]. 

**Figure 4:**
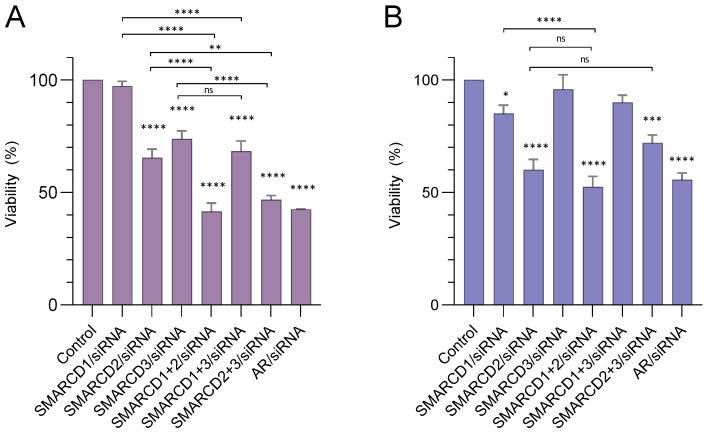
Effects of siRNA-mediated silencing of SMARCD1, SMARCD2 and SMARCD3 in various combinations on cell viability. siRNA-mediated knockdown of SMARCD1, SMARCD2 and SMARCD3 alone or in various combinations was performed in the cell lines (**A**) LnCAP or (**B**) C4-2 and cell viability was determined after five days. If not indicated otherwise by brackets, asterisks indicate the statistical significance of differences in cell viability between the respective sample and the negative control. Error bars indicate the standard error of the mean (SEM). Standard deviations were calculated using the relative cell viability determined in various biological replicates (*n* = 7). * *p* ≤ 0.05; ** *p* ≤ 0.01; *** *p* ≤ 0.001; **** *p* ≤ 0.0001; ns: not significant.

While the silencing of SMARCD1 did not cause significant effects in LnCAP cells, reduced SMARCD2 and SMARCD3 expression resulted in decreased cell viability (Figure 4A). The strongest decrease was observed due to simultaneous knockdown of SMARCD2 in combination with either of its paralogues (Figure 4A). In androgen-insensitive C4-2 cells, knockdown of SMARCD1 caused a mild decrease of cell viability, while SMARCD3/siRNA had no effects (Figure 4B). The strongest decrease of cell viability was observed in response to SMARCD2/siRNA; however, other than in LnCAP cells, this effect was not enhanced by simultaneous knockdown of SMARCD1 or SMARCD3 (Figure 4B).

**Figure 5:**
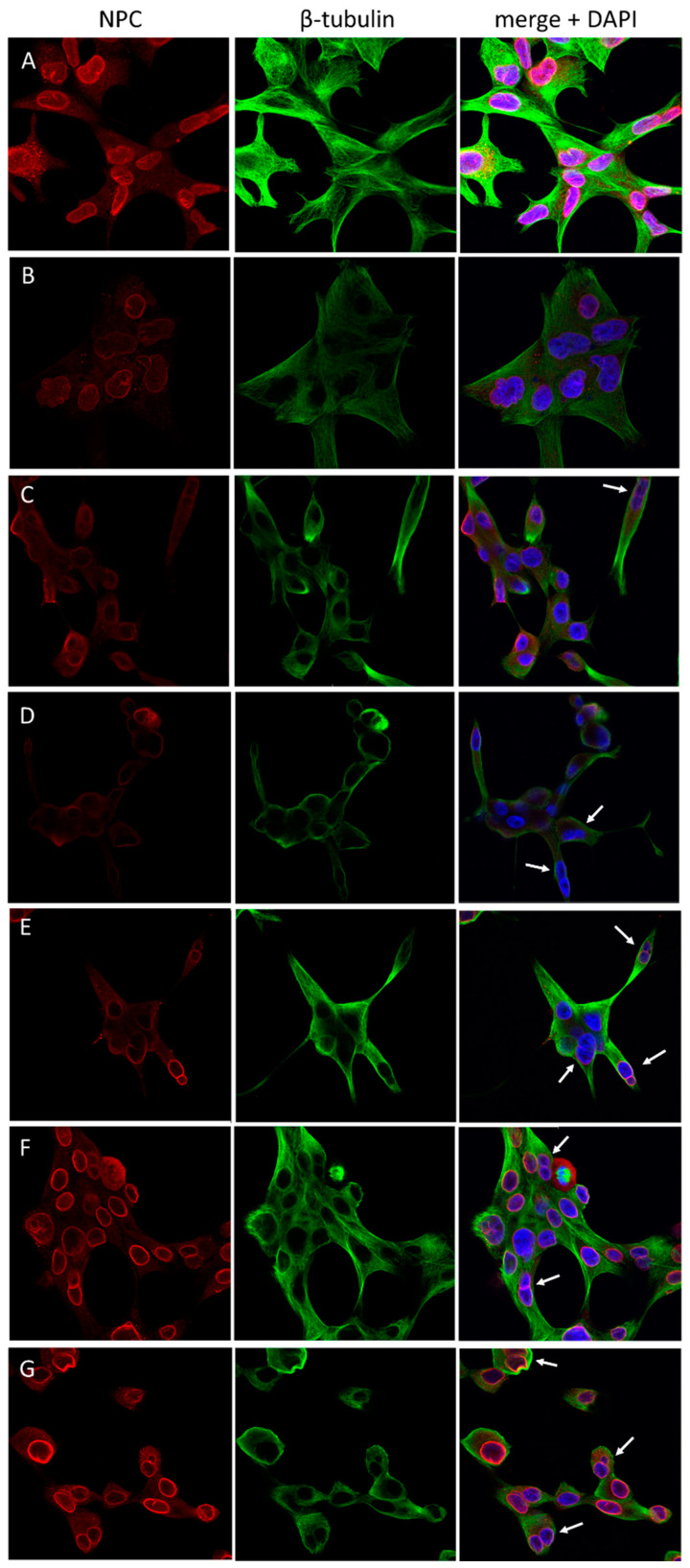
Morphologic effects of siRNA-mediated knockdown of SMARCD1, SMARCD2 and SMARCD3 in LnCAP cells. Immunostaining of β–tubulin and nuclear pore complex (NPC) proteins was performed in LnCAP cells treated with (**A**) a scrambled control or siRNAs targeting (**B**) SMARCD1, (**C**) SMARCD2, (**D**) SMARCD3, (**E**) SMARCD1 and SMARCD2, (**F**) SMARCD1 and SMARCD3 and (**G**) SMARCD2 and SMARCD3. Arrows indicate binucleated cells.

**Figure 6:**
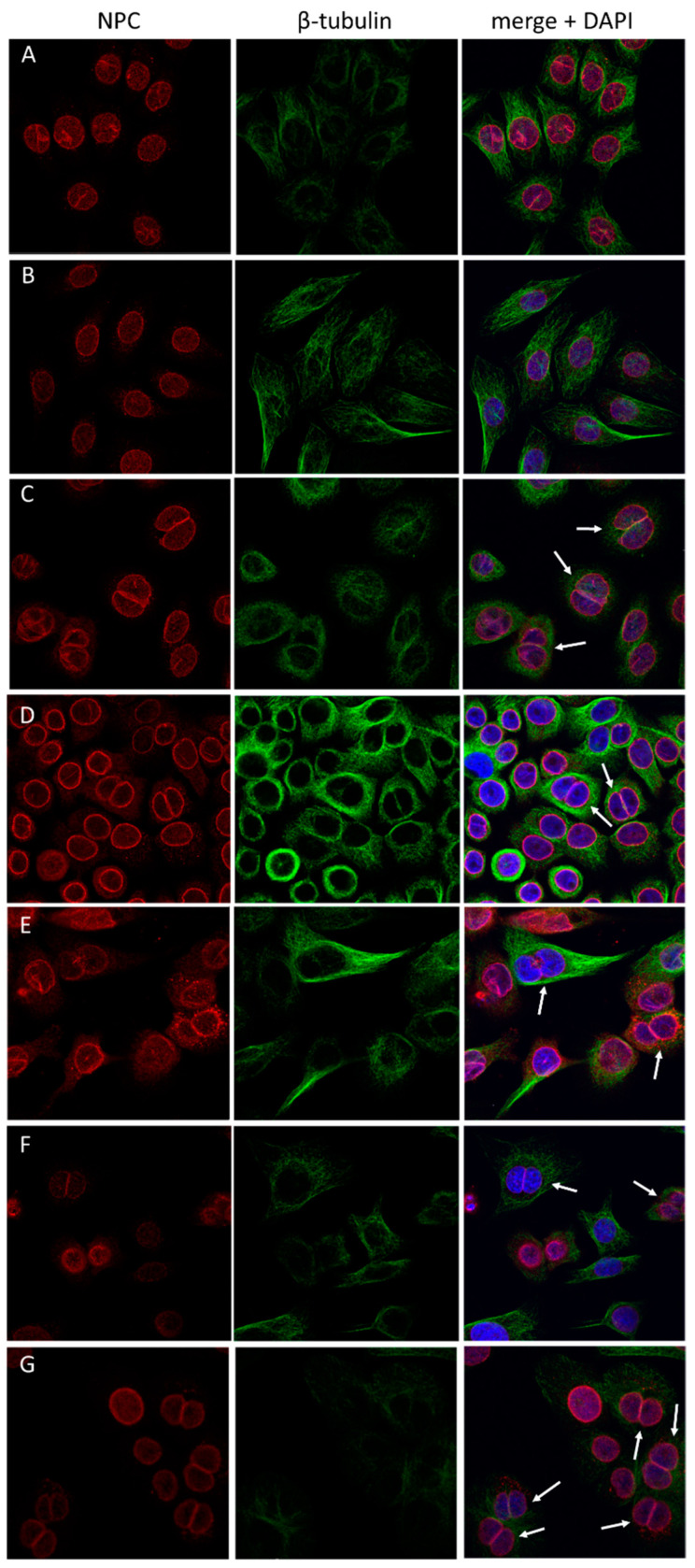
Morphologic effects of siRNA-mediated knockdown of SMARCD1, SMARCD2 and SMARCD3 in RWPE-1 cells. Immunostaining of β–tubulin and nuclear pore complex (NPC) proteins was performed in RWPE-1 cells treated with (**A**) a scrambled control or siRNAs targeting (**B**) SMARCD1, (**C**) SMARCD2, (**D**) SMARCD3, (**E**) SMARCD1 and SMARCD2, (**F**) SMARCD1 and SMARCD3 and (**G**) SMARCD2 and SMARCD3. Arrows indicate binucleated cells.

siRNA-mediated knockdown of the SMARCD genes also caused morphologic changes of LnCAP and RWPE-1 cells (Figure 5 and Figure 6). In LnCAP, SMARCD1/siRNA had a relatively mild impact and predominantly affected the nuclear shape (Figure 5B). Knockdown of SMARCD2 or SMARCD3, in contrast, had various severe phenotypic consequences including a diminuation of the cytoplasm, spindle-like morphologies and the formation of binucleated cells (Figure 5C,D). The latter phenotype was clearly enhanced upon simultaneous silencing of SMARCD2 with either of its paralogues (Figure 5E,G).

Similar to LnCAP, SMARCD1/siRNA influenced the nuclear shape of RWPE-1 cells, but had comparably mild overall effects (Figure 6B). Knockdown of SMARCD2 or SMARCD3 resulted in diminished cytoplasm and binucleated cells, whereby the latter effect was more pronounced than in LnCaP (Figure 6C,D). Simultaneous knockdown of SMARCD1 and SMARCD2 caused, amongst others, spindle-like morphologies (Figure 6E). Silencing of the SMARCD genes in either combination resulted in binucleated cells at high penetrance, whereby this effect was most pronounced when SMARCD2/siRNA and SMARCD3/siRNA was conducted in parallel (Figure 6E–G).

### 3.5. SMARCD1, SMARCD2 and SMARCD3 Are Involved in the Transcriptional Regulation of AR-Target Genes

To identify genes regulated by either of the SMARCD proteins, RNA-Seq was performed following siRNA-mediated knockdown of each individual paralogue. To distinguish genes that are regulated in a hormone-dependent manner, the approach was performed in LnCAP cells cultivated with or without physiologic levels of DHT.

**Figure 7:**
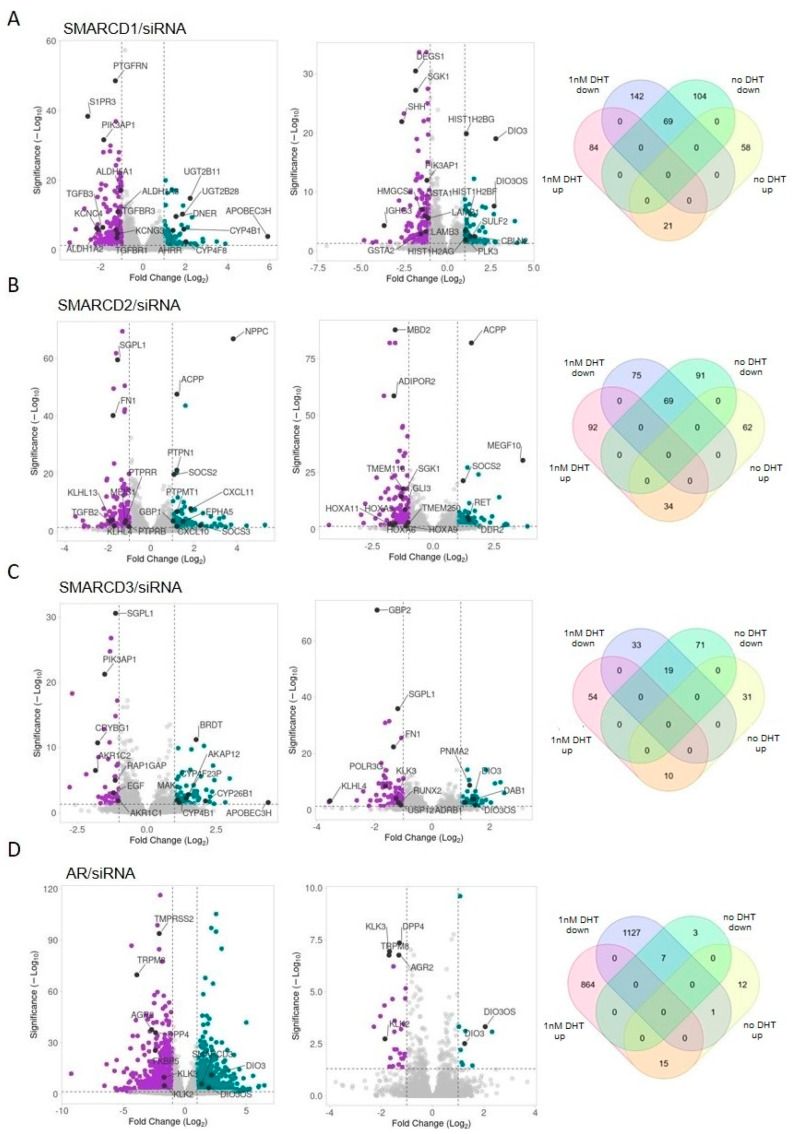
Differentially regulated genes in response to siRNA-mediated silencing of the SMARCD genes and AR performed in the presence or absence of DHT. siRNA-mediated knockdown of (**A**) SMARCD1, (**B**) SMARCD2, (**C**) SMARCD3 and (**D**) AR was performed in LnCAP cells grown in the presence (left) or absence (center) of DHT. RNA-seq was performed using RNA isolated from several biological replicates (*n* = 3). Differential gene expression was considered statistically significant for genes with log2 fold changes > ± 1 and adjusted *p*-values < 0.05. Genes exhibiting differential regulation exclusively in the presence or absence of DHT or under both experimental conditions were assessed (right).

Silencing of SMARCD1 in the presence of 1 nM DHT resulted in significant up- and down-regulation of 105 and 211 genes, respectively (Figure 7A, left and Table S1). Differentially regulated genes included *PTGFRN*, *S1PR3*, *PIK3AP1*, *APOBEC3H, DNER, AHRR*, genes encoding UDP glucuronosyltransferases (*UGT2B11*, *UGT2B28*), the cytochrome P450 superfamily (*CYP4B1*, *CYP4F8*), potassium voltage-gated channels (*KCNC4*, *KCNG3*), aldehyde dehydrogenases (*ALDH1A2*, *ALDH1A3*, *ALDH5A1*) and components of TGF signaling (*TGFBR1*, *TGFBR3*, *TGFB3*) (Figure 7A, left and Table S1).

In androgen-deprived cells, SMARCD1/siRNA caused increased expression of 79 and downregulation of 173 genes (Figure 7A, center; Table S1). Amongst others, *DEGS1*, *SGK1*, *SHH*, *SULF2*, *PLK3*, *IGHG3, PIK3AP1*, *HMGCS2, LAMB1*, *LAMB3*, *GSTA1*, *CBLN2*, *DIO3*, *DIO3OS* and several histone genes *(HIST1H2BG, HIST1H2AG*, *HIST1H2BF*) were differentially expressed (Figure 7A, center and Table S1). Comparing SMARCD1 downstream targets identified in the presence (*n* = 316) or absence (*n* = 252) of DHT, we found that 71.5% and 64.3%, respectively, exhibited differential regulation only under the given experimental condition (Figure 7A, right and Table S1). 

In response to SMARCD2/siRNA performed with DHT, we found 126 up-regulated and 144 downregulated genes (Figure 7B, left and Table S1). These comprised, e.g., *GBP1*, *NPPC*, *ACPP*, *FN1*, *MEIS1, EPHA5*, *SOCS2, SOCS3*, *TGFB2*, *SGPL1*, various genes encoding for C-X-C Motif Chemokine Ligands (*CXCL10, CXCL11),* Protein Tyrosine Phosphatases (*PTPMT1*, *PTPN1*, *PTPRB*, *PTPRR*) and Kelch-like family members (*KLHL4*, *KLHL13*) (Figure 7B, left and Table S1). 

In androgen-deprived cells, SMARCD2/siRNA caused increased expression of 96 genes, while 160 were significantly downregulated (Figure 7B, center and Table S1). Amongst others, *MBD2*, *RET*, *DDR2*, *ADIPOR2, SGK1, GLI3, ACPP*, *MEGF10*, *SOCS2*, genes encoding members of the homeobox A cluster family *(HOXA5*, *HOXA6*, *HOXA9*, *HOXA11*) and transmembrane proteins (*TMEM116*, *TMEM250*) were differentially expressed (Figure 7B, center and Table S1). As with SMARCD1, 61.9% and 59.8% of genes deregulated due to SMARCD2/siRNA in the presence (*n* = 270) or absence (*n* = 256) of DHT, respectively, showed differential regulation exclusively in the given experimental setting (Figure 7B, right and Table S1).

SMARCD3/siRNA performed with DHT resulted in up-regulation of 64 and down-regulation of 52 genes (Figure 7C, left and Table S1). Differentially regulated genes included *APOBEC3H*, *SGPL1*, *PIK3AP1*, *MAK*, *AKAP12*, *BRDT*, *EGF*, *AKR1C1*, *AKR1C2*, *RAP1GAP*, *CRYBG1* and various genes encoding members of the cytochrome P450 superfamily (*CYP26B1*, *CYP4B1*, *CYP4F23P*) (Figure 7C, left and Table S1). 

Under androgen-depleted conditions, SMARCD3/siRNA caused increased expression of 41 and downregulation of 90 genes (Figure 7C, center and Table S1). These genes comprised, e.g., *GBP2*, *SGPL1*, *FN1*, *KLK3*, *ADRB1*, *DAB1*, *DIO3*, *DIO3OS*, *PNMA2*, *POLR3G*, *RUNX2*, *KLHL4* and *USP12* (Figure 7C, center and Table S1). 75% and 77.9% of genes differentially regulated in the presence (*n* = 116) or absence (*n* = 131) of DHT, respectively, exhibited altered expression exclusively under the given experimental condition (Figure 7C, right and Table S1). Comparing downstream targets of SMARCD1, SMARCD2 and/or SMARCD3, we observed mainly independent, but also common regulatory functions of the SMARCD family members (Figure S6).

### 3.6. SMARCD Proteins Are Involved in the Transcriptional Regulation of AR-Regulated Genes 

Besides a highly conserved SWIB/MDM2 domain, all SMARCD proteins contain FxxLF-like and LxxLL motifs that mediate direct interactions with AR (Figure S4) [17,44]. To evaluate whether, besides SMARCD1, its paralogues may also represent AR cofactors, we sought to identify common downstream targets. AR/siRNA performed with physiological levels of 1nM DHT caused up- or down-regulation of 1135 and 879 genes, respectively (Figure 7D, left; Table S1). Importantly, we identified well-established direct AR targets, (e.g., *KLK3*, *TMPRSS2*, *FKBP5*), thereby validating our approach (Figure 7D, left and Table S1) [41,42,45]. In accordance with our previous findings, SMARCD3 was significantly upregulated (Figure 7D, left and Table S1). Given that AR is the key regulator of androgen response, it is not surprising that we identified only 38 differentially regulated genes in androgen-deprived cells (Figure 7D, center and Table S1). Genes exhibiting altered expression under both experimental conditions (*n* = 23) included *KLK2*, *KLK3*, *DPP4*, *TRPM8*, *AGR2, DIO3* and *DIO3OS* (Figure 7D, right; Table S1). 

Of the genes differentially regulated due to SMARCD1/siRNA in the presence of DHT (*n* = 316), 37% accounted for AR downstream targets (Figure S7A; Table S1); 66.7% of these genes exhibited the same expression patterns in response to SMARCD1/siRNA and AR/siRNA, and the remaining 33.3% were regulated in an antagonistic manner (Figure S7A and Table S1). Interestingly, also 36.1% of genes exhibiting altered expression levels due to SMARCD1/siRNA in androgen-deprived cells (*n* = 252) were found to be hormone-dependent AR targets; 53.8% of these genes were commonly regulated by SMARCD1 and AR (Figure S7A and Table S1). 

Similarly to SMARCD1, 38.9% and 35.9% of genes deregulated due to SMARCD2/siRNA performed with (*n* = 270) or without DHT (*n* = 256), respectively, were also regulated by AR (Figure S7B and Table S1). Most of these genes (w/ DHT: 67.6%; w/o DHT: 60.9%) exhibited the same expression pattern in response to knockdown of SMARCD2 and AR (Figure S7B and Table S1). Also SMARCD3 downstream targets identified in both the presence (*n* = 116) and absence of DHT (*n* = 131) exhibited large overlaps with AR-regulated genes (52.6% and 49.6%, respectively). The vast majority (w/ DHT: 72.1%; w/o DHT: 84.6%) was commonly regulated by SMARCD3 and AR (Figure S7C and Table S1).

Pathway analysis of AR-regulated genes revealed an enrichment of KEGG signaling pathways including “Steroid hormone biosynthesis”, “ECM-receptor interaction”, “Mucin type O-Glycan biosynthesis”, “ascorbate and aldarate metabolism” and “metabolism of xenobiotics by cytochrome P450” (Table S2). Several of these pathways were also found enriched analyzing genes deregulated in response to SMARCD1/siRNA, SMARCD2/siRNA and/or SMARCD3/siRNA performed with and without DHT (Table S2). Gene Set Variation Analysis (GSVA) of our RNAseq data confirmed alterations of androgen-related signaling pathways in response to AR/siRNA (Figure S8 and Table S3). GSVA further revealed an involvement of SMARCD1, SMARCD2 and SMARCD3 in the regulation of various androgen-dependent processes, in both, the absence and presence of hormones (Figure S8 and Table S3). As a further validation of our siRNA/RNA-seq approach, we confirmed the differential regulation of selected genes, most of which were previously shown to be involved in prostate tumorigenesis by, qPCR (Figure S9) [46,47,48,49,50,51,52,53,54,55,56].

## 4. Discussion

Previous studies suggest that SMARCD1 represents a direct, oncogenic AR-cofactor that regulates specific AR targets in a hormone-dependent manner [17,18]. In accordance with this finding, our RNA-seq approach revealed an overlap of PCa-related genes regulated by both SMARCD1 and AR in response to DHT (e.g., *TGFB3, TRPM8, PTGR1*) [53,56,57]. Similarly to SMARCD1, we found that numerous downstream targets of SMARCD2 (e.g., *PTGR1, TRMP8*, *PGC*) and SMARCD3 (e.g., *EGF*, *PIK3AP1*, *HSD3B1*) were AR-driven genes that are involved in prostate tumorigenesis, progression and metastasis [51,55,56,58,59,60]. Since all SMARCD proteins contain FxxFF and LxxLL motifs that mediate direct interactions with AR, we hypothesized that SMARCD2 and SMARCD3 also act as AR-cofactors [17,44]. The large overlap of SMARCD2- or SMARCD3-regulated downstream targets with AR-driven genes support this assumption. However, in order to gain a comprehensive understanding of SMARCD functions, we performed DHT induction for 24 h, thereby also identifying indirect targets of AR [39,40]. Thus, our approach does not prove that SMARCD2 and SMARCD3 also represent AR-cofactors directly participating in the transcriptional regulation of AR target genes, but clearly demonstrates an involvement of the SMARCD family in AR-driven pathways. Moreover, our RNA-seq approach showed that SMARCD1, SMARCD2 and SMARCD3 execute regulatory functions antagonizing AR, and are able to regulate subsets of genes in an hormone-independent manner. 

To inhibit AR-signaling, metastatic PCa patients are treated with androgen-deprivation therapies (ADT), that significantly extend overall survival [61]. Nevertheless, almost all patients eventually experience disease progression to castration-resistance, in which tumor cells grow and metastasize despite systemic castrate levels of androgens [62]. Previous studies have shown that castration-resistant prostate cancers (CRPC) remain addicted to AR signaling and that various mechanisms such as AR upregulation, activating AR mutations and overexpression of co-activators fuel AR hyperactivity [60]. Beyond that, alternative AR splice variants can cause reactivation of AR signaling in CRPC [63]. The most common and, therefore, best characterized variant AR-V7 was previously shown to regulate transcriptional targets that are divergent to those of full-length AR (fAR) [64]. AR-V7 inhibits a specific set of tumor suppressor genes, thereby contributing to castration-resistance [64]. Since AR-V7 variant lacks the ligand binding domain (LBD) it is constitutively active and resistant to currently available medications for CRPC such as abiraterone and enzalutamide [65]. To circumvent this issue, drugs that are able to induce the degradation of both fAR and AR-V7 are presently being developed [66]. The galeterone analog VNPP433-3β, for instance, was shown to be highly effective in in vivo models for CRPC and to have the ability to inhibit PCa stem cells [66,67]. Our RNA-seq approach revealed that SMARCD1, SMARCD2 and SMARCD3 affected the expression of highly divergent gene sets in the presence or absence of DHT. However, we identified numerous AR-driven genes that are regulated by the SMARCD proteins under both experimental conditions. Although in LnCAP cells AR-V7 is hardly expressed on a protein level, we suggest that interactions with other constitutively active AR variants may provide an explanation for this finding [68,69,70].

Interestingly, siRNA-mediated silencing of SMARCD1 showed relatively mild effects on the cellular morphology of RWPE-1 and LnCAP cells, while knockdown of SMARCD2 and SMARCD3 caused obvious alterations. These phenotypic effects included a diminution of the cytoplasm, changes of the nuclear shape and spindle-like cells, of which the latter suggests epithelial-to-mesenchymal transition (EMT) [71]. The most predominant phenotype were cytokinesis defects indicated by the formation of binucleated cells [72]. A previous study demonstrated that one of the two SMARCD homologues of the round worm *C. elegans*, SWSN-2.2, is required for correct chromosome inheritance [73]. Beyond that, it was shown that the protein directly interacts with various nuclear envelope components and is indispensable for nuclear reassembly after mitosis [73]. Given their high evolutionary conservation, we suppose that the SMARCD family members have similar functions in human prostate cells and that their partial loss, therefore, results in faulty cell division and altered nuclear morphology. We further suggest that cytokinesis defects cause, at least in parts, the reduction of LnCAP cell viability in response to knockdown of SMARCD2 and SMARCD3. The observation that silencing of SMARCD1 does not cause reduced cell viability or binucleated cells, but enhances both phenotypes caused by SMARCD2/siRNA, supports our hypothesis and suggests redundant functions of the two proteins in this biological context.

Taken together and in congruence with the literature, our approach demonstrated that SWI/SNF complexes have important yet paradoxical functions in PCa. It further showed that each SMARCD family member executes highly specialized functions in prostate cells. The finding that SMARCD3 was the only SWI/SNF subunit found to be deregulated in response to AR/siRNA reflects its functional specificity. However, in agreement with their high evolutionary conservation, we also identified common transcriptional targets of the SMARCD proteins. The enhancement of phenotypic effects upon simultaneous knockdown further suggest redundant functions of the SMARCD family in specific contexts. 

Drugs targeting specific SWI/SNF proteins are presently being developed [13,74]. Our study highlights the complexity of the functional interplay between paralogue SWI/SNF subunits that must be taken into consideration to develop safe and effective therapies targeting SWI/SNF.

## Figures and Tables

**Table 1:** Origin and molecular feature of cell lines included in the study. The classical PCa cell line models LnCAP, C4-2, PC3 and DU145 and the non-malignant cell line RWPE-1 were included in the study. These cell lines represent various disease stages and differ in terms of androgen responsiveness and AR expression levels.

Cell Line	Origin	Androgen Responsiveness	AR Expression
RWPE-1	non-malignant epithelial prostate cells immortalized with HPV18 [33]	androgen responsive [33,34]	high [34]
LNCaP	lymph node metastasis [35]	androgen responsive [34,35]	high [34]
C4-2	LnCAP subline isolated from xenograft tumor of castrated mouse [36]	androgen-independent [34,36]	low [34]
DU145	brain metastasis [37]	androgen-independent [34,37]	none [34]
PC3	bone metastasis [38]	androgen-independent [34,38]	none [34]

## Data Availability

All data generated or analyzed during this study are included in this published article and its supplementary files.

## Supplementary Materials

The following supporting information can be downloaded at: https://www.mdpi.com/article/10.3390/cells12010124/s1, Figure S1: Alterations of the SMARCD genes in various human malignancies; Figure S2: SMARCD1, SMARCD2 and SMARCD3 mRNA levels in various prostate cell lines; Figure S3: Effects of AR/siRNA on SMARCD1, SMARCD2 and SMARCD3 expression; Figure S4: FxxLF-like and LxxLL peptide motifs are present in all SMARCD family members; Figure S5: Efficiency and specificity of siRNAs targeting SMARCD1, SMARCD2 and SMARCD3; Figure S6: Independent and common downstream targets of SMARCD1, SMARCD2 and/or SMARCD3; Figure S7: Common downstream targets of SMARCD1, SMARCD2 or SMARCD3 and AR; Figure S8: Gene Set Variation Analysis (GSVA) of RNA-seq data; Figure S9: Validation of RNA-seq data by qPCR. Table S1: SMARCD3/siRNA w/ DHT; Table S2: Source Database; Table S3: gs.
