# Supplementary material for: The SMARCD Family of SWI/SNF Accessory Proteins Is Involved in the Transcriptional Regulation of Androgen Receptor-Driven Genes and Plays a Role in Various Essential Processes of Prostate Cancer
Source: Cells. 2022 Dec 28;12(1):124. doi: 10.3390/cells12010124 (PMC9818446; doi:10.3390/cells12010124)
Supplement: Supplementary file 1 [file cells-12-00124-s001.zip › Supplementary Figures.pdf]

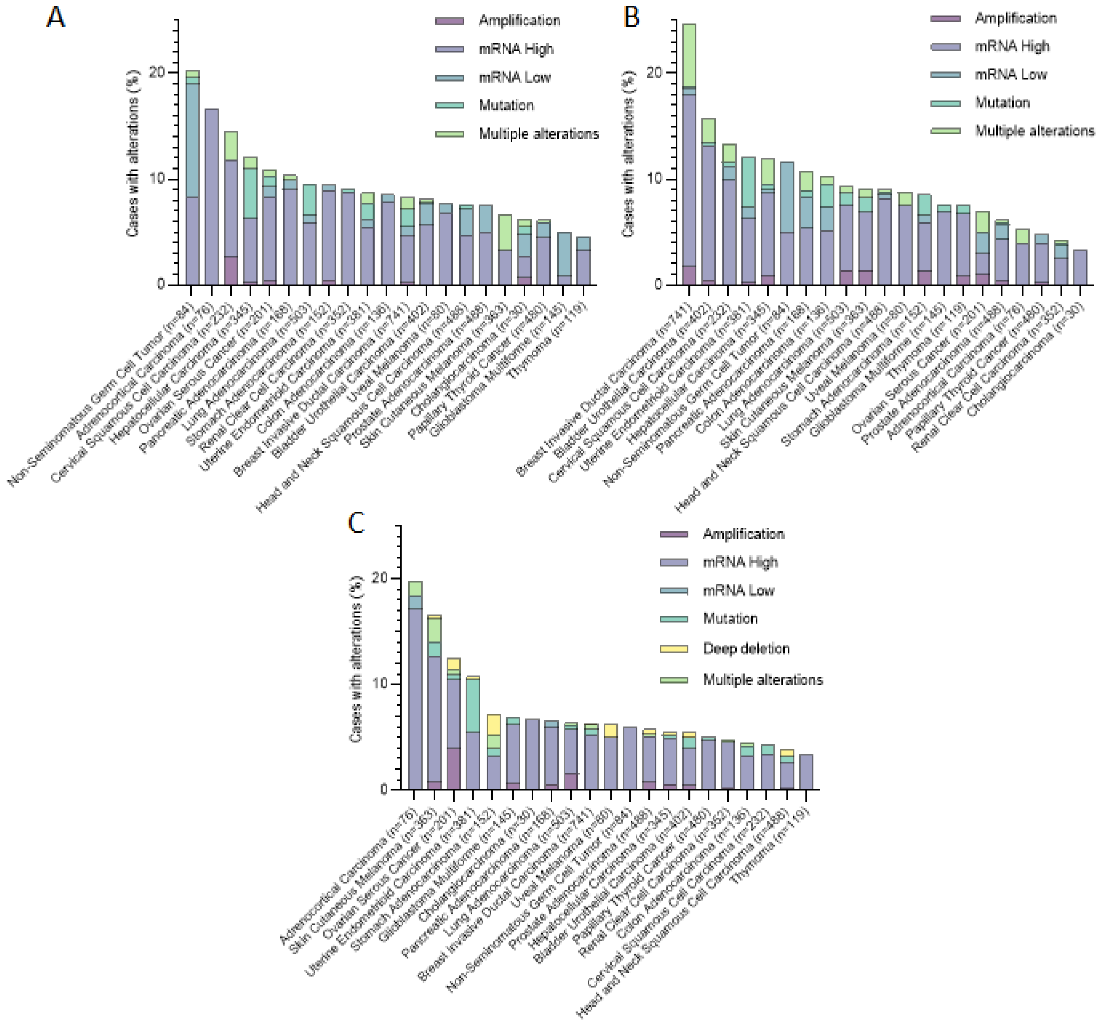

Suppl. Figure S1: Alterations of the SMARCD genes in various human malignancies

Compared to other human cancer types, mutations of **A)** SMARCD1, **B)** SMARCD2 and **C)** SMARCD3 are infrequent in PCa. As in other human malignancies, mRNA upregulation is the most common alteration.

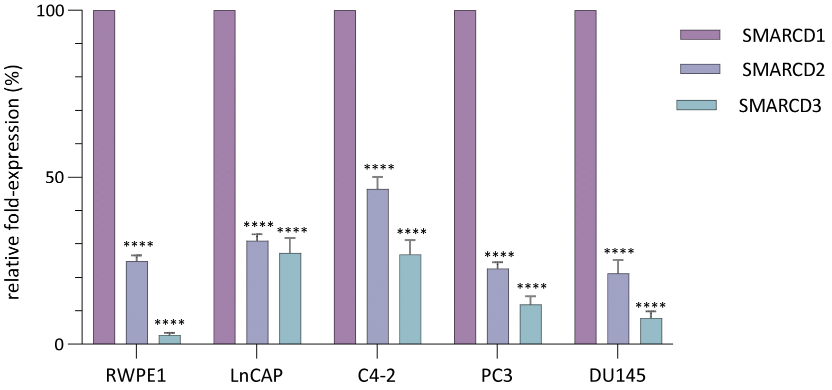

Suppl. Figure S2: SMARCD1, SMARCD2 and SMARCD3 mRNA levels in various prostate cell lines
mRNA expression of the SMARCD family members was assessed in non-malignant RWPE-1 cells and the PCa cell lines LnCAP, C4-2, PC3 and DU145. RNA was isolated from several biological replicates (n=3). Error bars indicate the standard error of the mean (SEM). Standard deviations were calculated using the relative expression determined in various technical replicates (n=6). Asterisks indicate the statistical significance of differences in SMARCD2 and SMARCD3 expression levels compared to SMARCD1. * $p \leq 0.05$; ** $p \leq 0.01$**;** *** $p \leq 0.001$**;** **** $p \leq 0.0001$; ns: not significant.

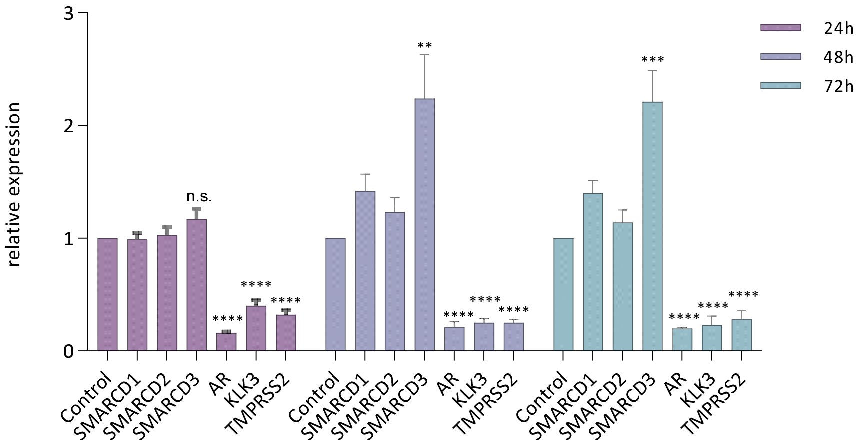

Suppl. Figure S3: Effects of AR/siRNA on SMARCD1,SMARCD2 and SMARCD3 expression
AR/siRNA was performed for 24h, 48h and 72h, resulting in a decrease of AR levels to 16%, 20% and 21%, respectively. Error bars indicate the standard error of the mean (SEM). RNA was isolated from several biological replicates (n=3). Standard deviations were calculated using the relative expression determined in various technical replicates (n=5). Asterisks indicate the statistical significance of differences in expression levels compared to the respective control. * $p \leq 0.05$; ** $p \leq 0.01$**; ***** $p \leq 0.001$**;** **** $p \leq 0.0001$; ns: not significant.

```
SMARCD1 isoform A      MAA--RAGF--QSVAPSGGAGASGGAGAAAALGPGGTPGPPVR-MGPAPGQGL-------
SMARCD1 isoform B      MAA--RAGF--QSVAPSGGAGASGGAGAAAALGPGGTPGPPVR-MGPAPGQGL-------
SMARCD2 isoform 1      --------------------------------------------MG--RRVGVEVTPRWA
SMARCD2 isoform 2      MSGRGAGGFPLPPLSPGGGAVAAALGAPPPPAGPGMLPGPALRGPGPAGGVGGPGAAAFR
SMARCD2 isoform 3      ------------------------------------------------------------
SMARCD3 isoform 1      -----------------------------------------------------------M
SMARCD3 isoform 2      --------------------------------------------MAADEVAGG--ARKAT

SMARCD1 isoform A      -YRSPMPGAAYPRPGMLPGSRMTPQGPSMGPP---GYGGNPSVRPGLAQSGMDQSRKRPA
SMARCD1 isoform B      -YRSPMPGAAYPRPGMLPGSRMTPQGPSMGPP---GYGGNPSVRPGLAQSGMDQSRKRPA
SMARCD2 isoform 1      PQ--KCQGARPQRPGMSPGNRMPMAGLQVGPPAGSPFGAAAPLRPGMPPTMMDPFRKRLL
SMARCD2 isoform 2      PMGPAGPAAQYQRPGMSPGNRMPMAGLQVGPPAGSPFGAAAPLRPGMPPTMMDPFRKRLL
SMARCD2 isoform 3      ---------------MSPGNRMPMAGLQVGPPAGSPFGAAAPLRPGMPPTMMDPFRKRLL
SMARCD3 isoform 1      TPGLQHPPTVVQRPGMPSGARMPHQGAPMGPP-GSPYMGSPAVRPGLAPAGMEPARKRAA
SMARCD3 isoform 2      KSKLFEFLVHGVRPGMPSGARMPHQGAPMGPP-GSPYMGSPAVRPGLAPAGMEPARKRAA

SMARCD1 isoform A      PQQIQQVQ------QQAVQNRNHNAKKKKMADKILPQRIRELVPESQAYMDLLAFERKLD
SMARCD1 isoform B      PQQIQQVQ------QQAVQNRNHNAKKKKMADKILPQRIRELVPESQAYMDLLAFERKLD
SMARCD2 isoform 1      VPQA----------QPPMPAQRRGLKRRKMADKVLPQRIRELVPESQAYMDLLAFERKLD
SMARCD2 isoform 2      VPQA----------QPPMPAQRRGLKRRKMADKVLPQRIRELVPESQAYMDLLAFERKLD
SMARCD2 isoform 3      VPQA----------QPPMPAQRRGLKRRKMADKVLPQRIRELVPESQAYMDLLAFERKLD
SMARCD3 isoform 1      PPPGQSQAQSQGQPVPTAPARSRSAKRRKMADKILPQRIRELVPESQAYMDLLAFERKLD
SMARCD3 isoform 2      PPPGQSQAQSQGQPVPTAPARSRSAKRRKMADKILPQRIRELVPESQAYMDLLAFERKLD

SMARCD1 isoform A      QTIMRKRLDIQEALKRPIKQKRKLRIFISNTFNPAKSDAED-----------GEGTVASW
SMARCD1 isoform B      QTIMRKRLDIQEALKRPIKQKRKLRIFISNTFNPAKSDAED-----------GEGTVASW
SMARCD2 isoform 1      QTIARKRMEIQEAIKKPLTQKRKLRIYISNTFSPSKAEGDSAGTAGTPGGTPAGDKVASW
SMARCD2 isoform 2      QTIARKRMEIQEAIKKPLTQKRKLRIYISNTFSPSKAEGDSAGTAGTPGGTPAGDKVASW
SMARCD2 isoform 3      QTIARKRMEIQEAIKKPLTQKRKLRIYISNTFSPSKAEGDSAGTAGTPGGTPAGDKVASW
SMARCD3 isoform 1      QTIMRKRVDIQEALKRPMKQKRKLRLYISNTFNPAKPDAED-----------SDGSIASW
SMARCD3 isoform 2      QTIMRKRVDIQEALKRPMKQKRKLRLYISNTFNPAKPDAED-----------SDGSIASW

SMARCD1 isoform A      ELRVEGRLLEDSALSKYDATKQKRKFSSFFKSLVIELDKDLYGPDNHLVEWHRTATTQET
SMARCD1 isoform B      ELRVEGRLLEDSALSKYDATKQKRKFSSFFKSLVIELDKDLYGPDNHLVEWHRTATTQET
SMARCD2 isoform 1      ELRVEGKLLDDP-------SKQKRKFSSFFKSLVIELDKELYGPDNHLVEWHRMPTTQET
SMARCD2 isoform 2      ELRVEGKLLDDP-------SKQKRKFSSFFKSLVIELDKELYGPDNHLVEWHRMPTTQET
SMARCD2 isoform 3      ELRVEGKLLDDP-------SKQKRKFSSFFKSLVIELDKELYGPDNHLVEWHRMPTTQET
SMARCD3 isoform 1      ELRVEGKLLDDP-------SKQKRKFSSFFKSLVIELDKDLYGPDNHLVEWHRTPTTQET
SMARCD3 isoform 2      ELRVEGKLLDDP-------SKQKRKFSSFFKSLVIELDKDLYGPDNHLVEWHRTPTTQET

SMARCD1 isoform A      DGFQVKRPGDVNVRCTVLLMLDYQPPQFKLDPRLARLLGIHTQTRPVIIQALWQYIKTHK
SMARCD1 isoform B      DGFQVKRPGDVNVRCTVLLMLDYQPPQFKLDPRLARLLGIHTQTRPVIIQALWQYIKTHK
SMARCD2 isoform 1      DGFQVKRPGDLNVKCTLLLMLDHQPPQYKLDPRLARLLGVHTQTRAAIMQALWLYIKHNQ
SMARCD2 isoform 2      DGFQVKRPGDLNVKCTLLLMLDHQPPQYKLDPRLARLLGVHTQTRAAIMQALWLYIKHNQ
SMARCD2 isoform 3      DGFQVKRPGDLNVKCTLLLMLDHQPPQYKLDPRLARLLGVHTQTRAAIMQALWLYIKHNQ
SMARCD3 isoform 1      DGFQVKRPGDLSVRCTLLLMLDYQPPQFKLDPRLARLLGLHTQSRSAIVQALWQYVKTNR
SMARCD3 isoform 2      DGFQVKRPGDLSVRCTLLLMLDYQPPQFKLDPRLARLLGLHTQSRSAIVQALWQYVKTNR

SMARCD1 isoform A      LQDPHEREFVICDKYLQQIFESQRMKFSEIPQRLHALLMPPEPIIINHVISVDPNDQKKT
SMARCD1 isoform B      LQDPHEREFVICDKYLQQIFESQRMKFSEIPQRLHALLMPPEPIIINHVISVDPNDQKKT
SMARCD2 isoform 1      LQDGHEREYINCNRYFRQIFSCGRLRFSEIPMKLAGLLQHPDPIVINHVISVDPNDQKKT
SMARCD2 isoform 2      LQDGHEREYINCNRYFRQIFSCGRLRFSEIPMKLAGLLQHPDPIVINHVISVDPNDQKKT
SMARCD2 isoform 3      LQDGHEREYINCNRYFRQIFSCGRLRFSEIPMKLAGLLQHPDPIVINHVISVDPNDQKKT
SMARCD3 isoform 1      LQDSHDKEYINGDKYFQQIFDCPRLKFSEIPQRLTALLLPPDPIVINHVISVDPSDQKKT
SMARCD3 isoform 2      LQDSHDKEYINGDKYFQQIFDCPRLKFSEIPQRLTALLLPPDPIVINHVISVDPSDQKKT

SMARCD1 isoform A      ACYDIDVEVDDTLKTQMNSFLLSTASQQEIATLDNKIHETIETINQLKTQREFMLSFARD
SMARCD1 isoform B      ACYDIDVEVDDTLKTQMNSFLLSTASQQEIATLDN-------------------------
SMARCD2 isoform 1      ACYDIDVEVDDPLKAQMSNFLASTTNQQEIASLDVKIHETIESINQLKTQRDFMLSFSTD
SMARCD2 isoform 2      ACYDIDVEVDDPLKAQMSNFLASTTNQQEIASLDVKIHETIESINQLKTQRDFMLSFSTD
SMARCD2 isoform 3      ACYDIDVEVDDPLKAQMSNFLASTTNQQEIASLDVKIHETIESINQLKTQRDFMLSFSTD
SMARCD3 isoform 1      ACYDIDVEVEEPLKGQMSSFLLSTANQQEISALDSKIHETIESINQLKIQRDFMLSFSRD
SMARCD3 isoform 2      ACYDIDVEVEEPLKGQMSSFLLSTANQQEISALDSKIHETIESINQLKIQRDFMLSFSRD

SMARCD1 isoform A      PQGFINDWLQSQCRDLKTMTDVVGNPEEERRAEFYFQPWAQEAVCRYFYSKVQQRRQELE
SMARCD1 isoform B      ----------------KTMTDVVGNPEEERRAEFYFQPWAQEAVCRYFYSKVQQRRQELE
SMARCD2 isoform 1      PQDFIQEWLRSQRRDLKIITDVIGNPEEERRAAFYHQPWAQEAVGRHIFAKVQQRRQELE
SMARCD2 isoform 2      PQDFIQEWLRSQRRDLKIITDVIGNPEEERRAAFYHQPWAQEAVGRHIFAKVQQRRQELE
SMARCD2 isoform 3      PQDFIQEWLRSQRRDLKIITDVIGNPEEERRAAFYHQPWAQEAVGRHIFAKVQQRRQELE
SMARCD3 isoform 1      PKGYVQDLLRSQSRDLKVMTDVAGNPEEERRAEFYHQPWSQEAVSRYFYCKIQQRRQELE
SMARCD3 isoform 2      PKGYVQDLLRSQSRDLKVMTDVAGNPEEERRAEFYHQPWSQEAVSRYFYCKIQQRRQELE

SMARCD1 isoform A      QALGIRNT
SMARCD1 isoform B      QALGIRNT
SMARCD2 isoform 1      QVLGIRLT              FxxLF-like peptide motif
SMARCD2 isoform 2      QVLGIRLT
SMARCD2 isoform 3      QVLGIRLT              LxxLL motif
SMARCD3 isoform 1      QSLVVRNT
SMARCD3 isoform 2      QSLVVRNT              SWIB/MDM2 domain
```

Suppl. Figure S4: FxxLF-like and LxxLL peptide motifs are present in all SMARCD family members

The SMARCD family members share a similarity of 57-72% at amino acid level and contain a highly conserved SWIB/MDM2 domain. All SMARCD proteins harbor the FxxLF-like motif required for direct interactions with the ligand-binding domain of AR, as well as LxxLL motifs, which also mediate direct interactions with AR. Amino acids that are present in two or three SMARCD family members are indicated in orange or red, respectively.

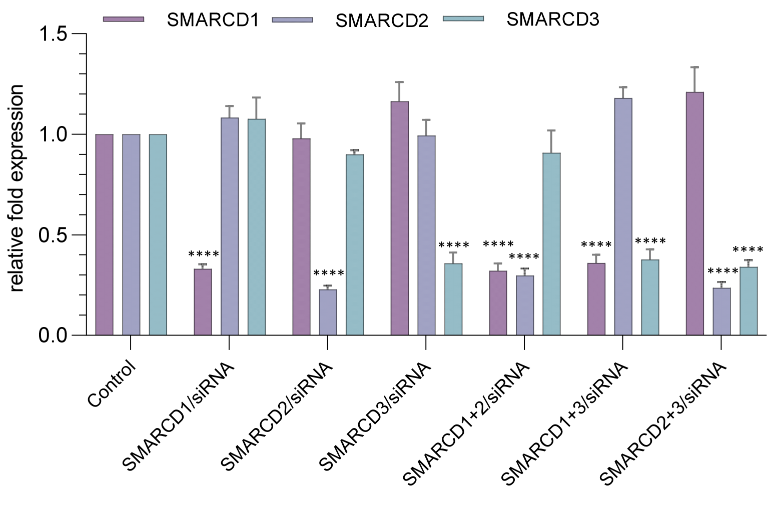

Suppl. Figure S5: Efficiency and specificity of siRNAs targeting SMARCD1, SMARCD2 and SMARCD3
siRNA-mediated knockdown of SMARCD1, SMARCD2 and SMARCD3 alone or in various combinations was performed in LnCAP cells and the expression levels of the three genes were determined by qPCR. RNA was isolated from several biological replicates (n=3). Asterisks indicate the statistical significance of differences in gene expression compared to the negative control. Error bars indicate the standard error of the mean (SEM). Standard deviations were calculated using the relative-fold expression determined in various technical replicates (n=6). * $p \leq 0.05$; ** $p \leq 0.01$**;** *** $p \leq 0.001$**;** **** $p \leq 0.0001$; ns: not significant.

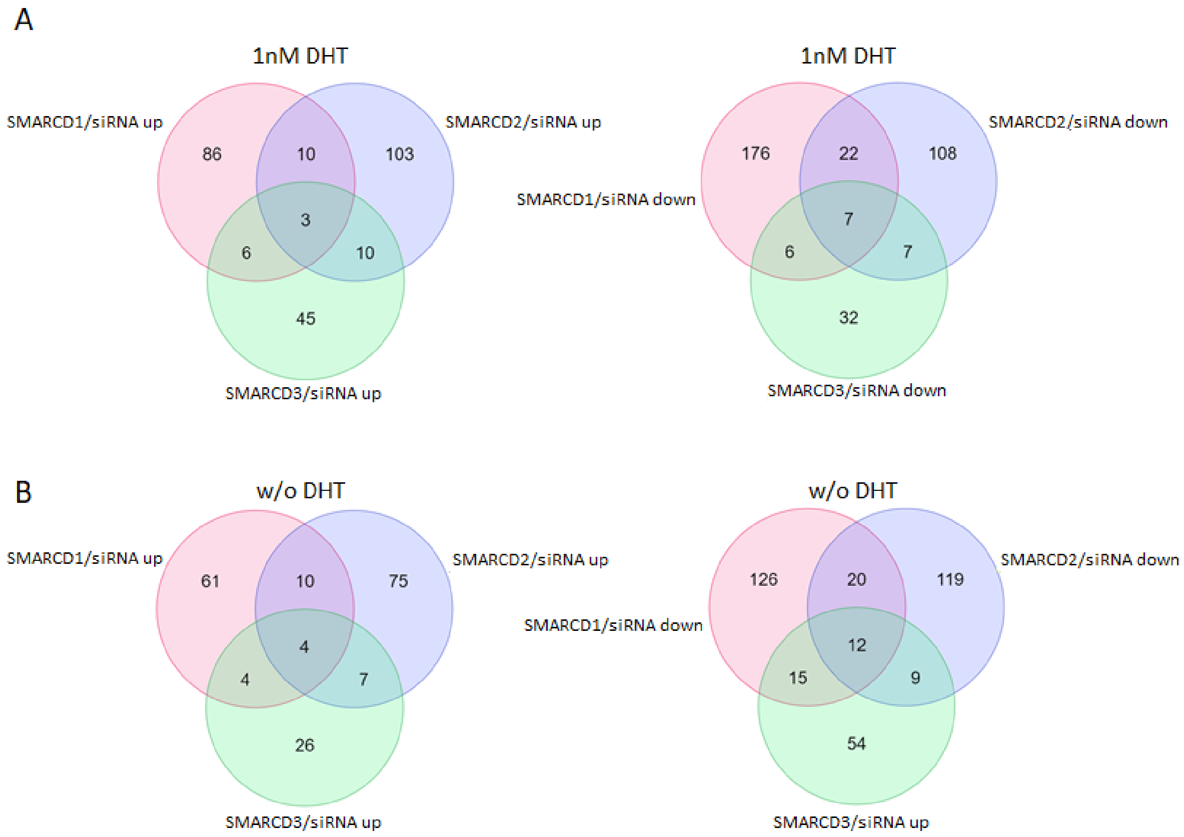

Suppl. Figure S6: Independent and common downstream targets of SMARCD1, SMARCD2 and/or SMARCD3 Genes regulated by SMARCD1, SMARCD2 and/or SMARCD3 in **A)** the presence or **B)** absence of hormones were identified by RNA-sequencing. Besides independent functions of SMARCD1, SMARCD2 and SMARCD3, various genes regulated by two or three members of the SMARCD family were identified.

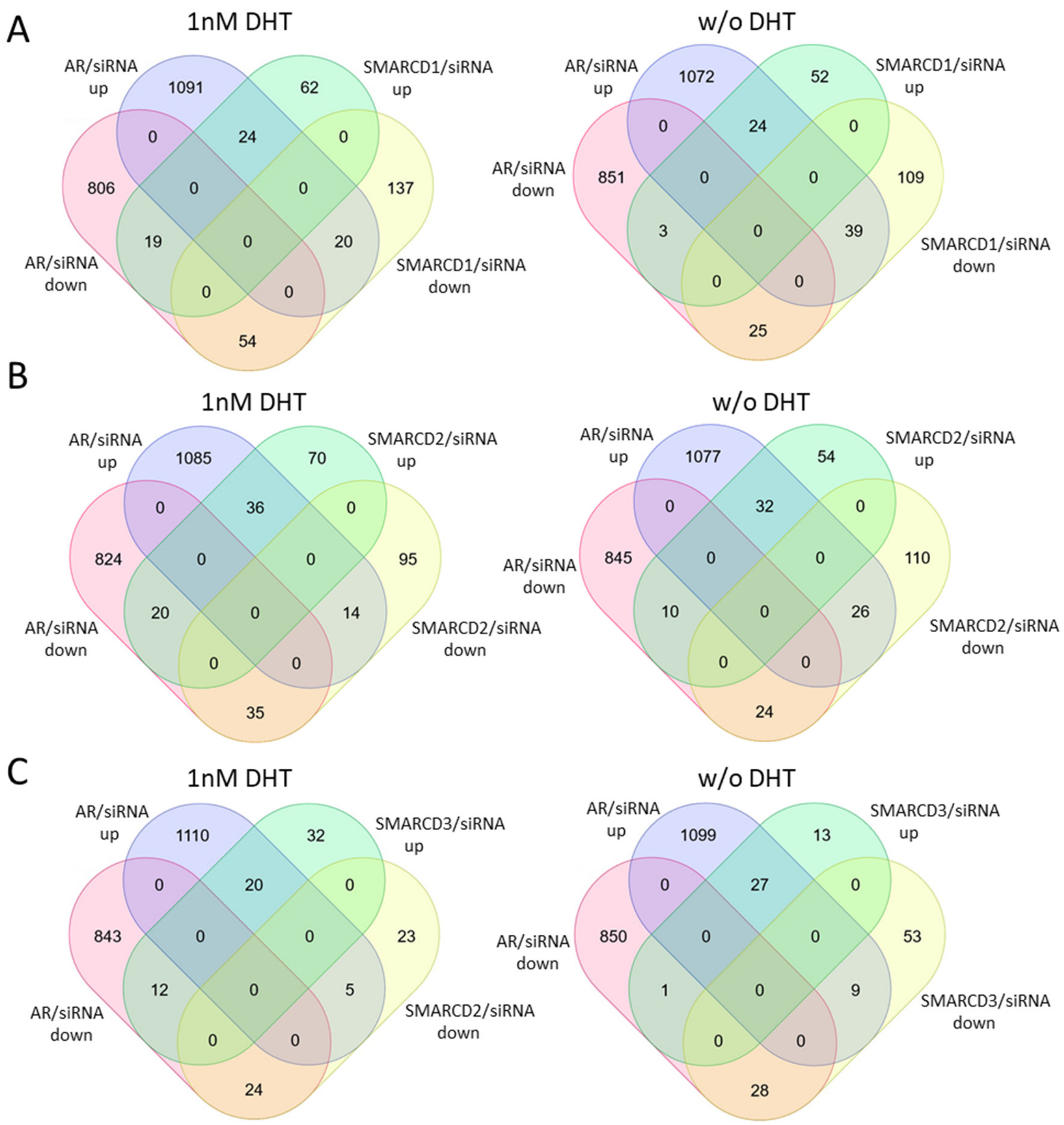

Suppl. Figure S7: Common downstream targets of SMARCD1, SMARCD2 or SMARCD3 and AR
Genes regulated by **A)** SMARCD1, **B)** SMARCD2 and **C)** SMARCD3 in the presence or absence of hormones were identified by RNA-sequencing and common downstream-targets with AR were assessed.

AR_DHT	AR_no DHT	SMARCD1_DHT	SMARCD1_no DHT	SMARCD2_DHT	SMARCD2_no DHT	SMARCD3_DHT	SMARCD3_no DHT	
-0.97	-0.23		-0.26		-0.14		-0.22	NELSON_RESPONSE_TO_ANDROGEN_UP
-0.97								WANG_RESPONSE_TO_ANDROGEN_UP
-0.71	-0.54	-0.57	-0.82	-0.41	-0.41	-0.47	-0.55	WP_ALTERNATIVE_PATHWAY_OF_FETAL_ANDROGEN_SYNTHESIS
-0.46						-0.17		DOANE_RESPONSE_TO_ANDROGEN_UP
	-0.75	-0.45	-0.47	-0.70	-0.56		-0.74	REACTOME_ANDROGEN_BIOSYNTHESIS
	-0.76	-0.66	-0.92	-0.51	-0.71			GOBP_ANDROGEN_BIOSYNTHETIC_PROCESS
			-0.45					GOBP_ANDROGEN_CATABOLIC_PROCESS
	-0.28						-0.29	REACTOME_ACTIVATED_PKN1_STIMULATES_TRANSCRIPTION_OF_AR_ ANDROGEN_RECEPTOR_REGULATED_GENES_KLK2_AND_KLK3
	-0.19	-0.25	-0.28	-0.33		-0.22	-0.21	GOBP_ANDROGEN_METABOLIC_PROCESS
				0.61	0.79		0.48	MOTAMED_RESPONSE_TO_ANDROGEN_DN
	0.34	-0.34						WP_ANDROGEN_RECEPTOR_SIGNALING_PATHWAY
	0.57		0.40				0.40	GOBP_REGULATION_OF_ANDROGEN_RECEPTOR_SIGNALING_PATHWAY
0.25		-0.20	-0.21					DOANE_RESPONSE_TO_ANDROGEN_DN
0.39	0.37		0.46		0.37			MOTAMED_RESPONSE_TO_ANDROGEN_UP
0.50								NELSON_RESPONSE_TO_ANDROGEN_DN

Suppl. Figure S8: Gene Set Variant Analysis (GSVA) of RNA-seq data

GSVA of RNA-seq data generated after siRNA-mediated knockdown of SMARCD1, SMARCD2 and SMARCD3 performed in the absence and presence of DHT revealed an involvement of the SMARCD proteins in various AR-driven pathways.

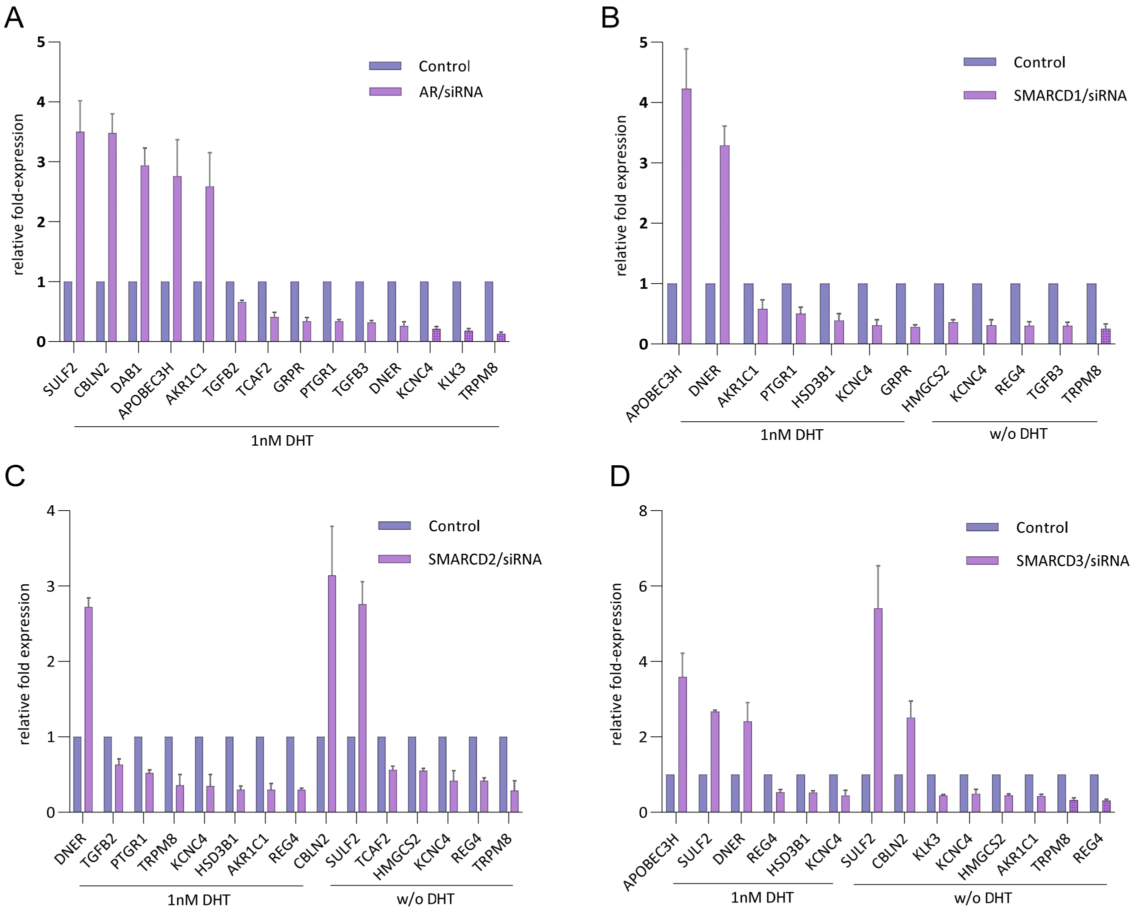

Figure S9: Validation of RNA-seq data by qPCR

To validate our RNA-seq approach, the differential regulation of genes identified as downstream-targets of **A)** AR, **B)** SMARCD1 **C)** SMARCD2 and **D)** SMARCD3 was verified by qPCR. RNA was isolated from several biological replicates (n=3). Error bars indicate the standard error of the mean (SEM). Standard deviations were calculated using the relative expression determined in various technical replicates (n=6). The differential regulation of all studied genes was statistically significant with p-values <0.001.
